# Herpetic meningoencephalitis and neurosyphilis – a rare association

**DOI:** 10.1186/1471-2334-14-S7-P34

**Published:** 2014-10-15

**Authors:** Liana-Cătălina Gavriliu, Elisabeta-Otilia Benea, Cosmina Andrei, Șerban Benea, Georgeta Ducu, Daniela Camburu, Alina Cozma, Roxana Dumitriu, Manuela Podani, Mihaela Ionică, Mădălina Simoiu

**Affiliations:** 1 National Institute for Infectious Diseases "Prof. Dr. Matei Balş", Bucharest, Romania; 2 Carol Davila University of Medicine and Pharmacy, Bucharest, Romania

## Background

A sudden onset of symptoms that sums up confusion and memory loss, associated with the characteristic cerebral MRI changes: T2 hyperintensity in mediotemporal area, are usually highly evocative for herpetic meningoencephalitis. The medical literature data describe cases of patients with symptoms and MRI changes that are typical for this infectious pathology, but that proved to be in fact cases of neurosyphilis mimicking herpetic meningoencephalitis.

## Case report

We describe the case of a patient hospitalized in INBI "Prof. Dr. Matei Balş", having clinical and imaging suspicion of herpetic meningoencephalitis, for which he promptly received acyclovir treatment and he had a spectacular favorable evolution. Because the patient’s family reported that the patient presented impaired memory and personality changes for several months, we decided to perform VDRL and TPHA quantitative tests from blood and cerebrospinal fluid (CSF) and the tests were positive. The patient was started on treatment for neurosyphilis, a week after acyclovir, as soon as we had the laboratory confirmation of the suspicions raised on the basis of the patient’s history. Unfortunately, due to some administrative difficulties, we couldn’t perform PCR for HSV from the CSF. However, the fact that the patient's evolution was favorable only with acyclovir, allows us to believe that we were in front of a rare combination of herpetic meningoencephalitis that occurred on an incidentally discovered neurosyphilis.

## Conclusion

To our knowledge, this may the first reported case of such an association. Neurosyphilis was confirmed on the basis of the specific tests performed from blood and CSF. Herpetic meningoencephalitis was highly suspected because of the symptoms, the MRI result and the spectacular improvement of the patient’s status only with acyclovir treatment. This case emphasizes the fact that an infectious pathology may not always be what it first seems to be, and that we should pursuit and clear any suspicion that we might have at some point.

